# Pediatric Restrictive Cardiomyopathies

**DOI:** 10.3389/fped.2021.745365

**Published:** 2022-01-25

**Authors:** Raffaello Ditaranto, Angelo Giuseppe Caponetti, Valentina Ferrara, Vanda Parisi, Matteo Minnucci, Chiara Chiti, Riccardo Baldassarre, Federico Di Nicola, Simone Bonetti, Tammam Hasan, Luciano Potena, Nazzareno Galiè, Luca Ragni, Elena Biagini

**Affiliations:** ^1^Cardiology Unit, Department of Experimental, Diagnostic and Specialty Medicine, IRCCS, Sant'Orsola Hospital, University of Bologna, Bologna, Italy; ^2^Pediatric Cardiac Surgery and GUCH Unit, IRCCS, Sant'Orsola Hospital, University of Bologna, Bologna, Italy

**Keywords:** cardiomyopathy, restrictive cardiomyopathy (RCM), sarcomeric cardiomyopathy, pediatric cardiomyopathies, heart transplant (HTx)

## Abstract

Restrictive cardiomyopathy (RCM) is the least frequent phenotype among pediatric heart muscle diseases, representing only 2.5–3% of all cardiomyopathies diagnosed during childhood. Pediatric RCM has a poor prognosis, high incidence of pulmonary hypertension (PH), thromboembolic events, and sudden death, is less amenable to medical or surgical treatment with high mortality rates. In this *scenario*, heart transplantation remains the only successful therapeutic option. Despite a shared hemodynamic profile, characterized by severe diastolic dysfunction and restrictive ventricular filling, with normal ventricle ejection fraction and wall thickness, RCM recognizes a broad etiological spectrum, consisting of genetic/familial and acquired causes, each of which has a distinct pathophysiology and natural course. Hence, the aim of this review is to cover the causes, clinical presentation, diagnostic evaluation, treatment, and prognosis of pediatric RCM.

## Introduction

Restrictive cardiomyopathy (RCM) is a heart muscle disease characterized by abnormal diastolic function with restrictive filling and normal ventricular size, wall thickness, and ejection fraction. Differently from hypertrophic, dilatated and right ventricle arrhythmogenic cardiomyopathy, where definition is based on morphology, RCM is defined based on physiology. However, under this common denominator, a wide spectrum of diseases are enclosed, with different causes, natural history, prognosis, and management. Furthermore, a restrictive hemodynamic profile can appear during the natural course of dilated and hypertrophic cardiomyopathy (HCM), being predictor of poor prognosis.

Among pediatric cardiomyopathies, RCM is the least common phenotype, representing only 2.5–3% of cardiomyopathies diagnosed during childhood (1). Unfortunately, compared to other pediatric cardiomyopathies, RCM is less amenable to medical or surgical treatment with higher mortality rates: 63% within 3 years of diagnosis and 75% within 6 years of diagnosis (2). As a consequence, rate for heart transplantation is relatively higher. Accordingly, within the Pediatric Heart Transplant Study Group, patients affected by RCM represents 12% of whole group of patients with cardiomyopathy undergoing heart transplantation.

Purpose of the present review is to summarize the causes of pediatric RCMs, their pathophysiology, clinical presentation and management.

## Definition and Epidemiology

According to European Society of Cardiology position statement, RCM is defined as a myocardial disease characterized by impaired ventricular filling and normal/reduced diastolic volumes in the presence of (near) normal ejection fraction and myocardial thickness (3). Decreased active relaxation and increased parietal stiffness cause pressure within the ventricles to rise precipitously during diastolic filling, with only small increases in volumes. Although systolic function was classically said to be preserved in RCM, contractility is rarely normal, indeed. Furthermore, restrictive physiology can also occur in other scenarios, namely end stage HCM and dilated cardiomyopathy (DCM). However, it is suggested to consider these two entities apart. Restrictive cardiomyopathy is the least common among pediatric cardiomyopathies, accounting for only 2–5% of all cases, although the incidence may be higher in tropical areas of Africa, Asia, and South America, where endomyocardial fibrosis (EMF) is endemic (4). Its prognosis is poor with a 2-year survival <50%, being heart transplantation the only effective treatment (5, 6). Restrictive cardiomyopathy has been described in children of all ages, with mean age at diagnosis ranging from 6 to 11 years old in recent studies.

## Etiology

**Figure 1:**
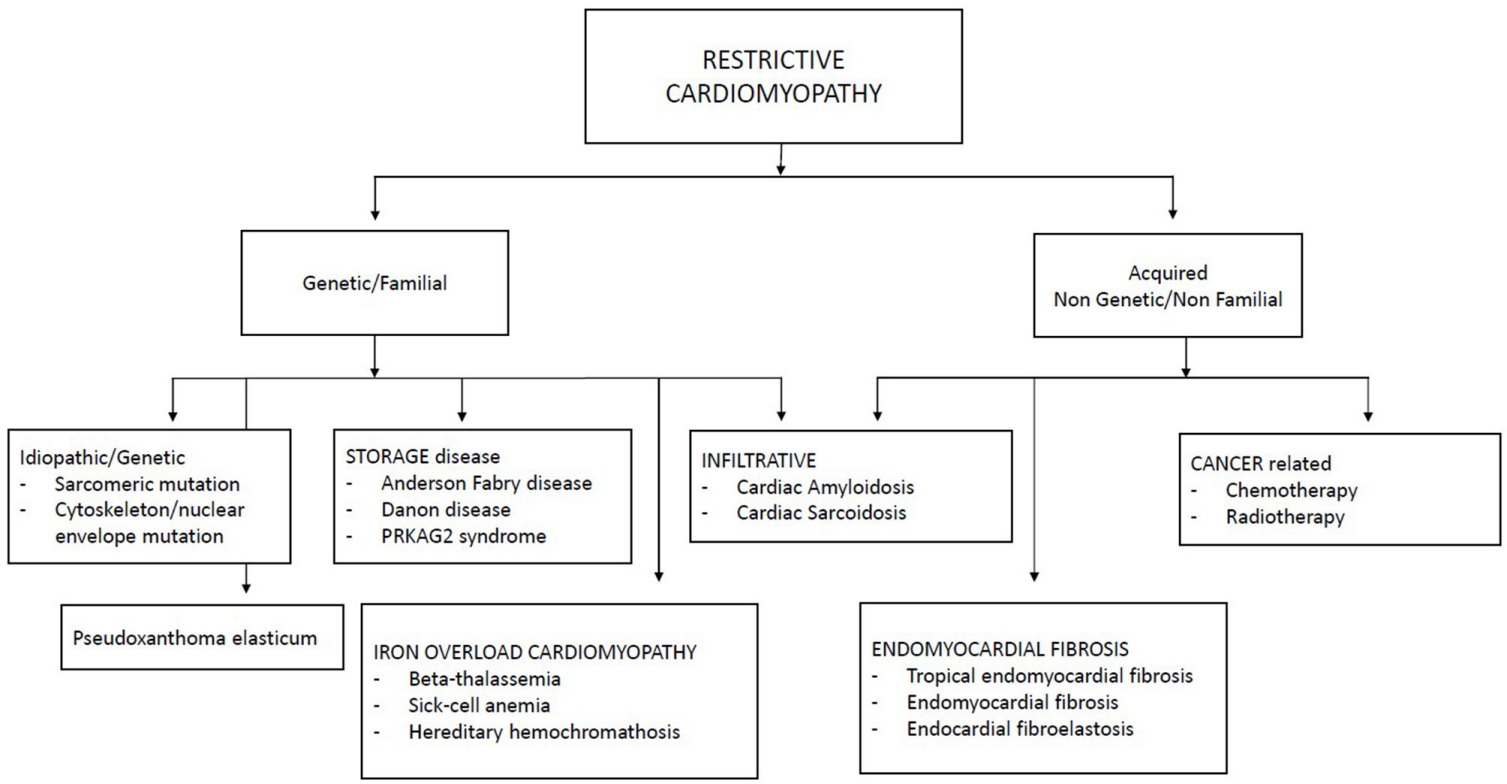
Classification of restrictive cardiomyopathies.

Restrictive cardiomyopathies represent a heterogeneous group of cardiomyopathies which recognize several etiologies, including inherited and acquired causes. The term of idiopathic RCM is probably no longer appropriate in a large group of patients: in fact, genetics has identified mutations in various genes, above all sarcomeric genes. Figure 1 summarizes the main causes of pediatric RCMs.

### Idiopathic/Genetic RCMs

Although in the past decades pediatric RCMs were most commonly considered idiopathic in origin, the technical progress in genetics and the introduction and diffusion of next-generation sequencing technology into clinical practice have broadened the genetic spectrum of RCMs, discovering disease-causing genes among affected children. The Pediatric Cardiomyopathy Registry Investigators, through whole-exome sequencing of 36 genes involved in cardiomyopathies, reported pathogenic or likely pathogenic variants in 50% of children with RCM (7). Furthermore, not infrequently, they found patients with multiple candidate causal alleles, suggesting that the interaction effects from several alleles may be clinically relevant in pediatric cardiomyopathies.

#### RCM Caused by Sarcomeric Gene Mutation

Hereditary sarcomeric contractile protein disease finds expression in a broad spectrum of phenotypes. In pediatric RCM, sarcomeric mutations represent the most frequently identified genetic defect, accounting for one-third of children with idiopathic RCM (8). Particularly the genes reported are: myosin-binding protein (*MYBPC3*), β-myosin heavy chain (*MYH7*), myosin light chain genes, titin *(TTN*), troponin I (*TNNI3*), troponin T (*TNNT2*), and α-cardiac actin (*ACTC*) (8–13).

**Figure 2:**
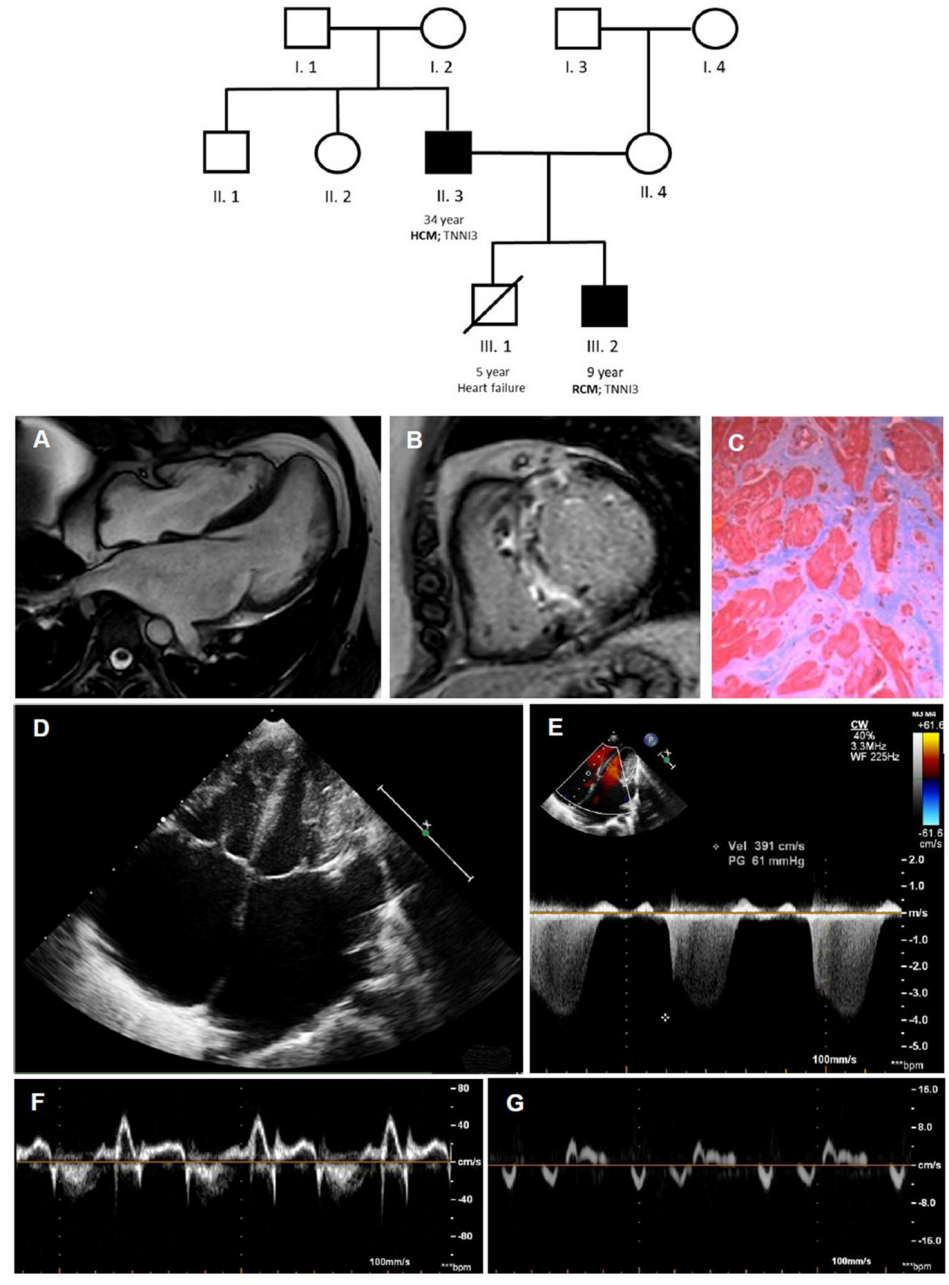
Coexistence of HCM [father; II.3; **(A–C)**] and RCM [son; III.2; **(D–G)**] within the same family, due to a pathogenetic troponin I mutation. In the family tree, black filled symbols stand for affected carriers. Cardiac magnetic resonance shows mild septum hypertrophy with mild left atrial enlargement **(A)** and septal late gadolinium enhancement **(B)** and replacement fibrosis at optical microscopy **(C)**. The echocardiogram **(D–G)** shows a pediatric RCM with severe biatrial enlargement and small left ventricular volume **(D)** with signs of increased filling pressures at Doppler and TDI evaluation. HCM, hypertrophic cardiomyopathy; RCM, restrictive cardiomyopathy.

Although the primary molecular pathways dysregulated in RCM are poorly understood, some hypotheses have been advanced in last years based on experimental models. Sarcomeric gene defects may increase Ca^2+^ sensitivity for force development, impair the inhibitory properties of troponin, activate thin-filament-mediated sarcomeric contraction at submaximal calcium concentrations, resulting in increased muscle tension during diastole and in abnormalities of cardiac relaxation (14). A striking variability in the phenotype, age of onset, and disease severity, even within the same family with a definite sarcomeric mutation, is often documented (Figure 2) (15). The basis of this phenotypic plasticity is unknown: probably is multifactorial and not solely dependent from the consequence of the mutated protein on the sarcomere structure/function (16). This suggests that—differently from a pure Mendelian inheritance disorder—a group of modifier genes, each exerting a modest effect, together with epigenetic, post-transcriptional, and translational modifications of expressed protein and environmental factors are responsible for phenotype definition (15). Therefore, the clinical phenotype of a genetic disorder is not simply determined by the information contained in the causal deoxyribonucleic acid sequence: this has relevant consequences, not only for pathophysiological understanding of cardiomyopathies but also to unravel molecular pathways to propel molecular based treatment strategies.

#### RCM Caused by Cytoskeletal/Nuclear Gene Mutation

Desmin, lamin, and filamin C mutations share a wide heterogeneity in clinical presentation and, particularly, the possibility to determine a RCM, sometimes associated with skeletal muscle involvement and atrio-ventricular conduction disturbances early in the course of disease.

**Figure 3:**
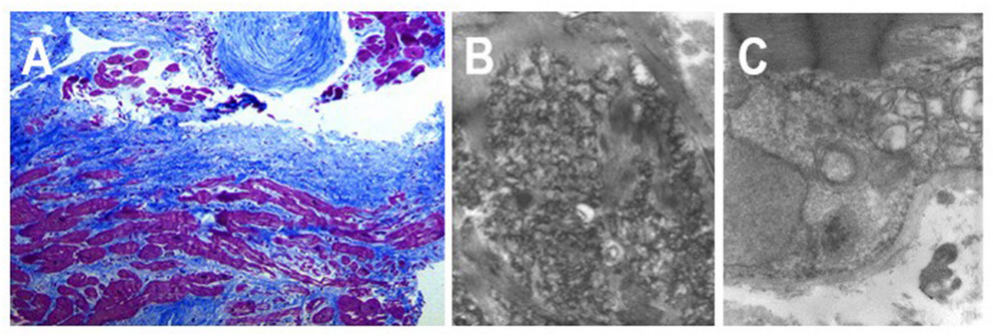
Endomyocardial biopsy from a 18 years-old patient affected by a desmin-related restrictive cardiomyopathy, carrying a pace-maker for complete atrio-ventricular block. **(A)** Histologic section shows severe interstitial fibrosis and morphological myocytes' abnormalities with cellular hypertrophy and cytoplasmic vacuolization. **(B,C)** Transmission electron micrographs show multiple deposits of granulofilamentous material in the intermyofibrillar region.

Desmin is a muscle-specific type III intermediate filament, important for the stability and correct cellular function, codified by *DES* gene (2q35). Notably, the spectrum of cardiac phenotypes associated with *DES* mutations ranges from dilated, arrhythmogenic, non-compaction, hypertrophic and, in rare cases, restrictive cardiomyopathies (17). Most of the known *DES* mutations are missense or small in-frame deletions. Many missense mutations introduce prolines that interfere with the hydrogen bonds within the peptide bonds of α-helices, thus destabilizing the protein structure. The majority of *DES* mutations are heterozygously inherited, indicating a dominant negative genetic mechanism or haploinsufficiency (18). However, a recessive autosomal transmission was reported in rare cases with compound heterozygous or homozygous *DES* truncating mutations (17). *DES-*related RCM may be associated with distal skeletal myopathy, atrio-ventricular blocks requiring pacemaker implantation and ventricular arrhythmias (19, 20). The ultrastructural characteristic is represented by granulofilamentous deposits in cardiac and skeletal muscle causing structural disorganization of the cytoskeleton leading to impairment of both myocyte relaxation and contraction (Figure 3).

Filamin C (or γ-filamin), coded by the *FLNC* gene (7q32.1), is member of a family of cross-link actin filaments expressed in cardiac and skeletal muscle, whose main role is to anchor membrane proteins to the cytoskeleton. Furthermore, filamin C is involved in protein degradation and autophagy control. The first association between RCM and *FLNC* mutations was described by Brodehl et al. in two unrelated Caucasian families with autosomal-dominant transmission, associated to atrial fibrillation and conduction disorders needing PM implantation (21). Cardiac histology showed myocytes hypertrophy with eosinophilic cytoplasmic aggregates due to mutated protein deposition, fibrosis, and mild disarray. Subsequent works by other groups reported families carrying *FLNC* missense mutations associated to variable degrees of a skeletal myofibrillar myopathy characterized by filamentous intracellular aggregates, combined with mild CK elevation, supraventricular arrhythmias, and RCM with early onset, often in childhood (22). Differently from missense mutation, *FLNC* truncating variants, were found in patients with a cardiac-restricted arrhythmogenic DCM phenotype characterized by a high risk of life-threatening ventricular arrhythmias (23).

Laminopathies are a heterogeneous group of diseases including heart disease, neuromuscular disorders, premature aging, and metabolic disorders, caused by mutation of *LMNA* gene (1q22), coding the nuclear envelope proteins lamin A and C, via alternate splicing. The spectrum of cardiac involvement ranges from supraventricular tachyarrhythmias and/or conduction system disease to DCM and ventricular tachyarrhythmias. Rarely *LMNA* can present as RCM in second decade of life, associated with atrio-ventricular blocks and requiring heart transplantation (24).

### Storage Cardiomyopathies

Among lysosomal storage disorders, Anderson Fabry disease (AFD), Danon disease, and PRKAG2 are the most frequently associated with cardiac involvement, generally presenting as HCM.

Anderson Fabry disease is caused by a reduced or absent activity of alpha-galactosidase A due to mutations in the *GLA* gene, mapping on X-chromosome (Xq22). This results in progressive globotriaosylceramide accumulation, in different cytotypes and tissues, with consequent organs dysfunction. Overt heart involvement is rare in childhood and may determine ECG abnormalities and initial diastolic dysfunction (25).

Danon disease is an X-linked multisystemic disorder caused by a defect in the lysosome-associated membrane protein 2 (*LAMP2*) gene (Xq24), encoding the LAMP2 protein, leading to progressive accumulation of autophagic material. Clinical phenotype is characterized by heart and skeletal myopathy, cardiac conduction abnormalities, mild intellectual difficulties, and retinal disease. Men are typically affected earlier and more severely than women. Cardiomyopathy had classically a hypertrophic phenotype, with high risk of end stage evolution and need for heart transplantation at early age (26).

PRKAG2 syndrome is a rare, early-onset autosomal dominant inherited glycogen storage disease, due to *PRKAG2* gene mutation (7q36.1), coding for the c subunit of the AMP-activated protein kinase. It is characterized by ventricular pre-excitation, supraventricular arrhythmias, and cardiac hypertrophy. It is frequently accompanied by chronotropic incompetence and advanced heart blocks, leading to premature PM implantation (27, 28).

### RCM Caused by Infiltrative Diseases

Amyloidosis is caused by deposition of an insoluble fibrillar protein called “amyloid” in the interstitium. It is extremely rare in childhood and is mostly seen late in life. Cardiac involvement is more commonly seen in transthyretin amyloidosis or light-chain amyloidosis, being mutated transthyretin amyloidosis (ATTRm) the earliest to occur in life, having been reported in the third decade of life (1).

Sarcoidosis is a multisystem, granulomatous disease of unknown etiology, characterized by non-caseating granulomas. Pediatric sarcoidosis is an extremely rare disease, with an estimated incidence of 0.6–1.02/100,000 children and a mean age at diagnosis of 11–13 years (29). Pediatric cardiac sarcoidosis is even rarer, though anecdotal cases were reported (30).

### Iron Overload Cardiomyopathy

Iron overload cardiomyopathy (IOC) results from iron accumulation as a consequence of excessive iron intake or absorption. Increased iron intake is generally caused by multiple red blood cell transfusions for chronic anemia (e.g., thalassemia, sickle cell disease, hemolytic anemias, inherited bone marrow failure syndrome, myelodysplastic syndrome) or less commonly by infusions of iron-containing products used to treat certain porphyrias. Increased iron absorption is mainly caused by hereditary hemochromatosis (HH), due to mutations in genes involved in iron metabolism, increasing gastro-intestinal absorption. Among the known four HH subtypes, type 2 (also called juvenile hemochromatosis) typically presents by the second decade with a more severe phenotype accompanied, in addition to cardiomyopathy, by hypogonadotropic hypogonadism, arthropathy, and liver fibrosis or cirrhosis. Type 2 HH can be caused by two different genes: *HFE2* gene mutation, encoding hemojuvelin, a protein that interacts with hepcidin (subtype 1A) or *HAMP* gene, codying for hepcidin, a key regulator of circulating iron (subtype 2B). Regardless of its origin, IOC is characterized by a RCM with prominent early diastolic dysfunction which progressively evolute to an end-stage DCM (31). Although immunoinflammatory and inherited component may contribute to cardiac injury, iron overload plays a central role in the pathophysiology of IOC. Iron toxicity has been attributed to the production of free oxygen radicals, as a result of free iron availability. Excessive circulating free iron (i.e., not bound to transferrin) enters the cardiomyocytes, mainly through voltage-dependent L-type Ca^2+^ channels, in the form of Fe^2+^ (ferrous iron). Inside the cardiomyocytes, free iron induces the formation of reactive oxygen species, hence causing peroxidative damage of cellular structures (lipids, proteins, nucleic acids), cellular apoptosis, and finally cardiac dysfunction (32). Furthermore, iron overload increases calcium influx, which might impair, in its turn, diastolic function. A post-mortem hearts series (comprising two children) from patients with severe cardiac siderosis and heart failure leading to death or heart transplantation, except the severe granular iron deposition on Perl's stain, did not reveal replacement fibrosis and minor interstitial fibrosis was also unusual and very limited in extent, underlining the potential reversibility of heart failure in IOC (33). The best validated method for quantifying myocardial iron overload *in vivo* is T2^*^ mapping with CMR. A T2^*^ value of <20 ms at 1.5 T, typically measured in the interventricular septum, is used as a conservative cut-off for segmental and global heart iron overload and patients with the lowest T2^*^ values have the highest risk of developing arrhythmia and heart failure (34).

### RCM Caused by Fibrotic Process

- Tropical EMF is the most common cause of RCM, affecting more than 12 million people worldwide. Initially described in Uganda, EMF is commonly reported in rural populations of equatorial developing countries, where exhibits a bimodal distribution, peaking at 10 and 30 years of age. Many etiological hypotheses, not mutually exclusive, have been proposed: malnutrition, parasitic infections, environmental factors, and genetics. Due to regional differences in disease prevalence, a geochemical basis has been advanced as unifying hypothesis (35). Despite different possible candidates (magnesium deficiency, cerium toxicity, cyanogenic glycosides, high vitamin D, serotonin toxicity, herbal preparations), for none of them definitive evidence is available. The natural history of EMF is characterized by recurrent hot phases with inflammation and eosinophilia, progressing to a chronic phase where a biventricular RCM, caused by deposition of fibrous tissue in the endomyocardium, affects both ventricles or less frequently exclusively the right ventricle. In this last case, chronic venous hypertension causes facial edema and exophthalmos, jugular venous distention, hepatomegaly, and ascites, often out of proportion to peripheral edema (36).
- Eosinophilic EMF is a rare cause of RCM, resulting from toxicity of eosinophils toward cardiac tissues in patients with a hypereosinophilic syndrome (HES). Although the causes for eosinophilic infiltration of myocardium are various (hypersensitivity, parasitic infestation, systemic diseases, myeloproliferative syndrome, and idiopathic HES), pediatric HES is commonly associated with chromosomal abnormalities, and in 40% of the cases, it has been associated with acute leukemia (37). Eosinophil-mediated heart damage evolves through three stages, although these stages may be overlapping and not clearly sequential. The *acute necrotic stage* is characterized by infiltration of eosinophils and release of their granules' contents in the myocardium (eosinophilic myocarditis). Thereafter, an *intermediate phase* follows, with thrombus formation along the damaged endocardium (more often in the apex of the left ventricle) and finally a *fibrotic stage* characterized by reduced ventricular compliance and RMC. At this stage, entrapment of the chordae tendineae can lead to mitral and tricuspid regurgitation (38).
- Endocardial fibroelastosis is a congenital disease characterized by diffuse thickening of the LV endocardium secondary to proliferation of fibrous and elastic tissue, leading to early death. It manifests with a DCM phenotype or with a RCM phenotype with a small LV cavity. Most forms of endocardial fibroelastosis are associated with congenital heart diseases, first of all hypoplastic left heart syndrome and aortic stenosis/atresia, but also coarctation of the aorta, patent ductus arteriosus and, mitral regurgitation. In the minority of cases, a familial recurrence is seen, with all possible pattern of inheritance reported (39). Despite various attempts to unravel its origin, a definite mechanism could not be identified. Genetic predisposition, viral infection (particularly mumps virus), and hypoxia during fetal cardiac development have been proposed as putative causes. Cardiac transplantation is required for end-stage heart failure (39). A promising surgical approach to remove endomyocardial layer showed improvement in the restrictive physiology together with growth of the left ventricle in parallel with somatic growth (40).
- Pseudoxanthoma elasticum is an inherited systemic disease of connective tissue, affecting skin, retina, and cardiovascular system. It is transmitted in an autosomal recessive manner and is caused by mutations in the *ABCC6* (ATP binding cassette subtype C number 6) gene (41). Histology of affected tissues exhibits elastic fiber mineralisation and fragmentation (so called “elastorrhexia”) (42). Restrictive cardiomyopathy in relation to diffuse endocardial fibroelastosis is very rare (43).

### Oncological Cardiomyopathy

Progress in cancer therapeutics over the past years has significantly improved survival rates for most childhood malignancies. Unfortunately, the developing cardiovascular system of children and adolescents is particularly vulnerable to most pediatric cancer protocols, relying on cytotoxic chemotherapy and radiation. Indeed, cardiac-specific disease is the most common non-cancer cause of death among long-term childhood cancer survivors, only second to the recurrence of primary cancer and the development of second cancers (44).

Anthracyclines (such as doxorubicin, daunorubicin, and epirubicin), used to treat hematologic cancers and solid tumors, are among the most used chemotherapeutic agents causing cardiotoxicity. Although the typical manifestation of cancer drug induced cardiomyopathy is a DCM, with LV dilation and thinning of myocardial wall, with “restrictive” physiology in the more advanced stages, a not negligible proportion of long-term survivors will eventually develop a RCM. Importantly, patients may present cardiotoxicity many years after treatment completion, needing carefully monitoring for years by echocardiography. However, despite the adverse cardiac effects of anthracyclines, these drugs are fundamental components and standard of care for many types of cancer. Risk factors identified for cardiotoxicity include: female sex, younger age at diagnosis, black race, trisomy 21, and certain lifestyle behaviors (1). Although a total cumulative anthracycline dose >300 mg/m^2^ was identified as significant risk factor for late-occurring anthracycline-induced cardiotoxicity, adverse effects were reported also with lower cumulative doses (45).

Radiotherapy is frequently used together with surgery/chemotherapy in thoracic malignancies and lymphomas. Cardiac exposure is generally due to “stray” radiation as the heart is almost never the actual target, except for rare sarcomas or metastases (46). Although modern planning and irradiation techniques have significantly improved, radiation induced cardiac injury represents an actual issue, and combination with chemotherapy and novel agents increase cardiac toxicity. Restrictive cardiomyopathy is the consequence of a diffuse biventricular fibrosis, most often with a non-ischemic pattern, which reduces myocardial compliance. However, coexistent radiation induced micro and macrovascular disease can result in ischemia/infarction and regional fibrosis.

In the next years the number of the long-term cancer survivors is expected to rise, not only for improved long-term survival rates but—unfortunately—also for the increased incidence of many histological subtypes of childhood cancer: consequently, amelioration of prevention and treatment strategies is needed.

## Emodinamics

Restrictive cardiomyopathy recognizes a unique hemodynamic profile, independently from the specific cause at the basis of diastolic dysfunction. In RCM impairment of diastole can be related both to the abnormal myocardial relaxation (i.e., the active actin-myosin cross-bridge detachment) and to the increased myocardial stiffness due to the myocardial cells (e.g., titin) and the interstitial matrix (fibrosis) alterations, determining elevated left and right-sided filling pressure. Although left ventricular ejection fraction is typically preserved, systolic contractility is often impaired as showed by tissue Doppler imaging and speckle tracking. Systo-diastolic dysfunction leads to reduced stroke volume. In the protodiastole—despite delayed active relaxation—there is an unusually early rapid filling of the ventricles, due to high atrial pressures, halted by incompliant ventricular walls from the end of the first third of diastole onward—reflecting myocardial stiffness. This results in a prominent “y” descent on the atrial pressure curves and, sometimes, in the *square root* or *dip and plateau sign* on ventricular pressure curves, consisting in an early decrease in ventricular diastolic pressure followed by a rapid rise to a plateau phase. During the following atrial contraction, the stiff ventricles are unable to easily accept additional blood volume, and thus the contribution from atrial contraction is often minimal. Differently from constrictive pericarditis (CP), in patients with RCM there is not enhanced ventricular interdependence, with concordant left and right ventricular pressures during the respiratory cycle and parallel changes in their pressure curve areas. Moreover, atria progressively enlarge, due to high intracavitary pressures and thin and distensible walls, predisposing to atrial arrhythmias and thromboembolic episodes. A relevant proportion of patients develop pulmonary hypertension (PH) with elevated pulmonary vascular resistances, unresponsive to vasodilator testing, precluding them form orthotopic heart transplantation (47).

## Clinical Presentation

The clinical presentation of RCM can be highly variable in children population, ranging from asymptomatic to right and/or left heart failure with PH.

Biventricular systolic function is typically normal until advanced stages of the disease, leading to heart failure with preserved ejection fraction (HFpEF). Consequently, clinical presentation is characterized by dyspnoea, poor appetite, ascites, peripheral edema, and hepatomegaly. However, while in adults with RCM symptoms and signs of heart failure are easy to detect, clinical evaluation in children is challenging because of the non-specific findings, resulting in some delay in correct diagnosis. Besides, children with RCM may have a history of frequent respiratory infections. Progressive atrial enlargement can also lead to atrial arrhythmias such as atrial fibrillation and thromboembolic complications, with mitral and tricuspid functional regurgitation frequently associated, due to anulus dilatation. Wolff Parkinson-White syndrome with supraventricular tachycardia has also been reported.

Sudden death occurs in ≈25% of pediatric RCM patients, with an annual mortality rate reported of 7%, being cardiac ischemia, arrhythmias, and thromboembolic events the main responsible. Various risk factors for sudden death in pediatric RCM have been identified in previous studies: cardiomegaly, thromboembolism, raised pulmonary vascular resistance, pulmonary venous congestion, syncope, chest pain, left atrial size, PR and QRS duration. Albeit inconsistently sometimes, the main limitation of these studies is their retrospective nature and the intrinsic bias associated. Rivenes et al. evaluated a cohort of 18 pediatric patients with RCM who had sudden, unanticipated cardiac arrests, identifying chest pain, and syncope as risk factors for sudden death (48). Histopathologic evidence for ischemia was found in the majority of patients who died and the evidence of ischemia at Holter monitoring (i.e., ST depression) predicted death within several months. They proposed lethal ventricular arrhythmias as cause of death, showing examples of ventricular tachycardia/fibrillation recorded during resuscitation attempts (48). Complementary, Walsh et al. in a 16 pediatric RCMs cohort reported five sudden cardiac events, with three patients having complete heart block. In one of them, ST-segment elevation was documented before the onset of complete heart block, suggesting an underlying ischemic process as trigger of the bradyarrhythmia. Older age at presentation, longer PR interval and QRS duration were associated with sudden cardiac events (49).

## Diagnosis

**Figure 4:**
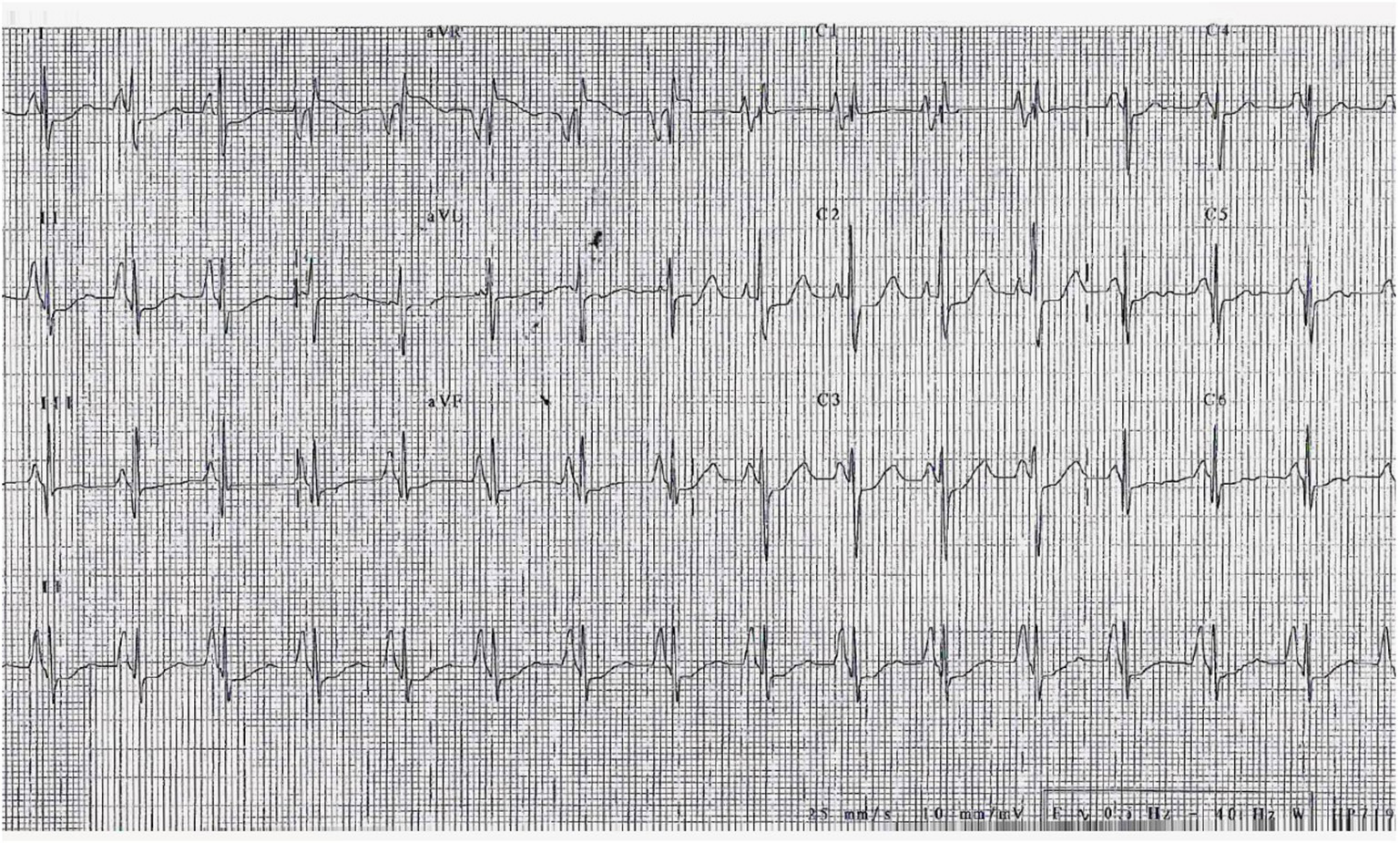
ECG of a 3-year-old girl, affected by restrictive cardiomyopathy, showing biatrial enlargement and diffuse repolarization abnormalities. In the peripheral leads, please note the *monstre* atrial enlargement.

Approximately 98% of RCM patients have an abnormal electrocardiogram (ECG) (50). The most common abnormalities are right and/or left atrial enlargement (91% of patients) (Figure 4). ST-T segment and T waves abnormalities are also frequently present and may be most evident at higher heart rates. In a small cohort of 12 children affected by RCM, Hayashi et al. found that obliquely elevated ST-T segments and notched or biphasic T waves were the most frequent ventricular repolarization abnormalities (67% of patients). Besides, the criteria for biventricular hypertrophy based on QRS voltage were achieved in seven patients (although three of them actually had some degree of hypertrophy at echocardiogram) (51). ST-T depression is usually mild and non-specific, but in some cases can be pronounced, mimicking a left main or proximal left anterior descending artery occlusion or a multivessel coronary disease. This was associated with high risk of sudden cardiac death (48) and Selvaganesh et al. hypothesized that marked ST depression can be caused by high end-diastolic pressure, impairing perfusion in the subendocardial region or stretching the myocardium and activating stretch sensitive channels (52). Conduction abnormalities can also be seen, as well as right or left ventricular hypertrophy signs (48). Furthermore, serial ECG-Holter monitoring is useful for the evaluation of rhythm disturbances and ST segment analysis.

Chest X-ray is abnormal in nearly 90% of cases and usually show cardiomegaly and pulmonary venous congestion (51).

**Figure 5:**
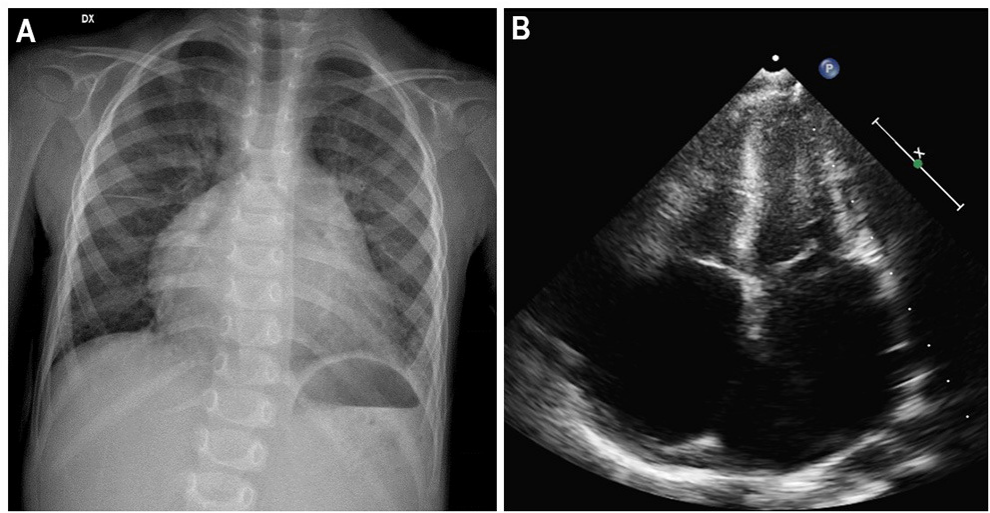
Three-year-old girl affected by restrictive cardiomyopathy. Antero-posterior chest radiograph **(A)** showing massive cardiomegaly due to severe biatrial enlargement, as confirmed by apical four-chamber echocardiography **(B)**.

Echocardiography plays a key role in RCM diagnosis. Marked biatrial enlargement with a normal or slightly decreased LV ejection fraction are widely considered as pathognomonic findings (Figure 5). Regardless of etiology, the considerable elevation in filling pressures in patients with RCM is reflected in abnormal mitral inflow and tissue Doppler variables. A short (<140 ms) mitral deceleration time, increased pulsed wave (PW) Doppler mitral E/A ratio (>2.5) and E/e′ >15 are markers of significantly elevated left filling pressures (53). Although IVRT (isovolumic relaxation time) is prolonged when myocardial relaxation is impaired, due to delayed LV pressure falling during the isovolumic relaxation, a short LV IVRT of <50 ms is frequently detected in RCM, as consequence of the high LA pressure (53). Typically plethoric inferior vena cava and hepatic veins are seen and, with inspiration, diastolic flow reversal in the hepatic veins is documented due to the inability of a non-compliant right ventricle to accommodate the increased venous return. Echocardiography is helpful in differential diagnosis between RCM and CP. Although both conditions shares E-wave predominance and short deceleration time, respirophasic shifting of the interventricular septum, caused by exaggerated interventricular dependence, is characteristic of CP. Accordingly respiratory flow variations consisting in increasing >25% in mitral inflow during expiration and >40% in tricuspid inflow after inspiration are absent in RCM but frequently noted in CP (54). However, among all echocardiographic parameters, the most useful to distinguish RCM from CP are those of tissue Doppler imaging. In fact a normal tissue Doppler e′ velocity (>8 cm/s) indicating normal LV relaxation virtually excludes RCM. In patients affected by CP, where diastolic dysfunction is due to pericardial constraint, e′ is normal or even increased since the longitudinal movement of the myocardium is enhanced because of constricted radial motion. Furthermore, in patients with pericardial constriction, lateral mitral annular e′ is usually lower than e′ from the medial annulus (so called “*annulus reversus*”) (55). This finding, absent in RCM, reflects the tethering of the lateral mitral annulus to the adjacent fibrotic and scarred pericardium. In this regard, speckle tracking may add even higher diagnostic accuracy in differentiating constriction from restriction: in fact while in RCM both radial and longitudinal strains are reduced due to a diseased myocardium, in CP reduction mainly involve circumferential strain, reflecting the subepicardial tethering offered by the fibrous pericardium (55, 56). The myocardial performance index (MPI) or Tei index may provide further information of both LV diastole and systole, with normal value of 0.33 ± 0.02 from 3 to 18 years old. It is defined as the sum of the isovolumic contraction and relaxation times divided by the ejection time, and it can be calculated from PW Doppler at the mitral and aortic valve simultaneously or using TDI at mitral valve annulus (57). Moreover, lateral a′ velocity (cut-off ≤ 0.042 m/s) and pulmonary vein A wave duration (cut-off ≥156 m/s) both have sensitivity and specificity ≥80% for LVEDP ≥ 20 mmHg measured on right heart catheterization (58). Ancillary methods such as pulmonary regurgitant flow velocity, color M-mode flow propagation, and myocardial velocity gradient have been proposed for differential diagnosis between RCM and CP, however data in pediatric age are lacking. Finally, the performance and interpretation of diastolic measurements in children are challenging, given the higher heart rates, potential need for sedation, together with conflicting and limited data on the relationships among the diastolic variables and the degree of dysfunction (1). Indeed, in the assessment of diastolic dysfunction among 175 children with cardiomyopathy, the percentage of normal diastolic variables in children with overt cardiac dysfunction was high, with discordance between e′ and left atrium (LA) volume criteria. Patients with RCM were best identified with mitral E deceleration time, which was found to be abnormal in 75% of patients (59). In another study, Sasaki et al. described the LA area indexed to body surface area as the most useful measurement to differentiate between healthy children and RCM patients (60).

Cardiac magnetic resonance (CMR) offers a better spatial resolution than echocardiography, providing detailed information about anatomic structures, ventricular function, perfusion, and tissue characterization (61). For instance, T2^*^-weighted CMR is the diagnostic gold standard to detect and quantify myocardial iron content in IOC (62). Late gadolinium enhancement (LGE) can show peculiar patterns of replacement and reactive fibrosis, which can direct the diagnosis to specific subtypes of RCM. In AFD, LGE is typically localized in the infero-lateral mid-basal wall of left ventricle, and because of the distinctive fatty nature of the intracellular deposits, native T1 mapping has typically a low value, in contrast to most of other infiltrative or storage cardiomyopathies (63). In cardiac sarcoidosis LGE distribution is patchy, often with multifocal distribution, not following a coronary artery topography, sparing the endomyocardial layer, and involving mainly the basal and lateral LV walls (64). When performing CMR, it must be considered that the young patients must hold still in the scanner and follow the instructions to minimize motion artifacts during image acquisition. Whereas, this is possible in older children (more than 6–8 years of age), it requires sedation and anesthesia for younger patients, with different possible strategies, often depending on institutional preference and availability of resources such as pediatric anesthesiologists (65).

Whole-body scintigraphy or SPECT with bone-seeking tracers [(99mTc)-labeled bisphosphonate compounds: pyrophosphate (PYP); 3,3-diphosphono-1,2-propanodicarboxylic acid (DPD), and hydroxydiphosphonate (HDP)] can reveal amyloid deposits (especially in ATTR subtype) in the heart, as well as PET with [^18^F] FDG can detect inflammatory cells in some pathological processes such as cardiac sarcoidosis. However, due to the rarity of the aforementioned diseases and due to the concern for radiation exposure in childhood, nuclear imaging has very limited applications in the diagnostic work-up of pediatric RCMs.

**Figure 6:**
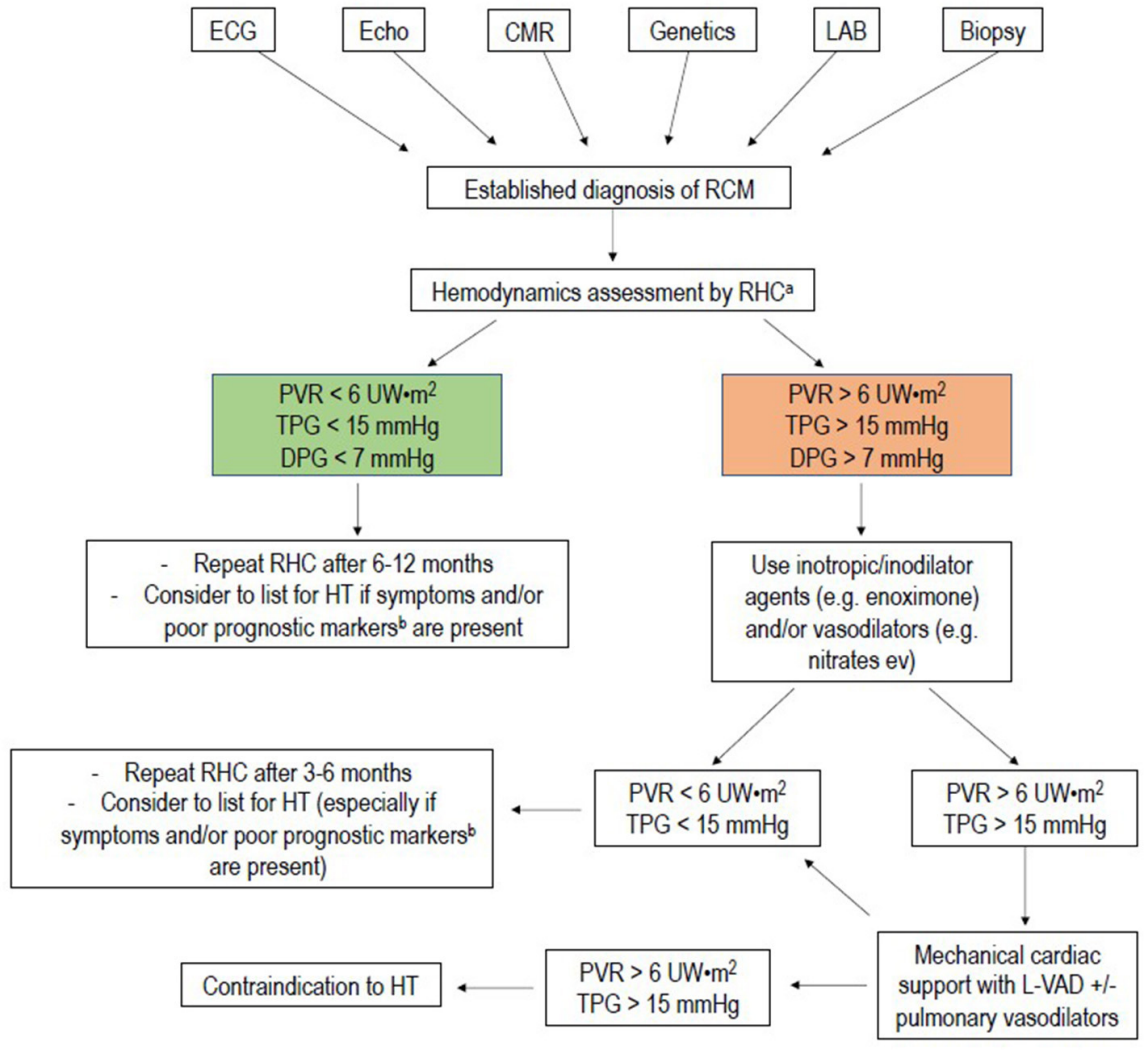
Flow-chart of management of pediatric restrictive cardiomyopathy. RHC, right heart catheterization; PVR, pulmonary vascular resistance; mPAP, mean pulmonary artery pressure; TPG, transpulmonary gradient; DPG, diastolic pulmonary gradient; HT, heart transplantation. ^a^RHC is usually indicated for all patients referred for heart transplantation. RHC in addition, may help to stratify prognosis in patients in NYHA class I to II: severe pulmonary hypertension and low cardiac output can develop even in absence of symptoms, leading to late referral for heart transplantation. When at least one of the prognostic indicators for RCM is present, it may be advisable to refer the patient to a tertiary center for HT evaluation. ^b^Poor prognostic markers are: pulmonary congestion, myocardial ischemia, severe left atrial dilation, male gender, reduction of left ventricular fractional shortening, increased left ventricular walls thickness.

Cardiac catheterization is usually not necessary for RCM diagnosis, but it can be useful to distinguish between restrictive and constrictive physiology and to determine the severity of the diastolic dysfunction by directly measuring the filling pressures of both ventricles (Figure 6). In RCM left ventricular end-diastolic pressures are usually higher than right end-diastolic pressure, whereas are equal or very nearly equal in CP. Furthermore, specular discordance between RV and LV peak systolic pressures during inspiration are typical of CP, with an increase in RV pressure occurring during peak inspiration, when LV pressure is lowest. Cardiac catheterization can also reveal the presence of PH, detect the presence of elevated pulmonary vascular resistance, evaluate the cardiac index and test for pulmonary vasculature reactivity.

Plasma levels of natriuretic peptides can be helpful in the diagnostic pathway of RCM, especially in the differential diagnosis with CP. A study of 49 adults (20 with RCM and 29 with CP) showed that median plasma NT-proBNP was 1,775 (208–7,500) pg/ml in those with RCM vs. 124 (68–718) pg/ml in those with CP (*P* = 0.001) (66). Specific etiologies may require additional laboratory exams such as angiotensin converting enzyme dosage in sarcoidosis, complete blood count to establish eosinophilia in HESs, serum iron concentrations, total iron-binding capacity, and ferritin levels in hemochromatosis, alpha-galactosidase activity, and lyso-Gb3 levels in AFD, immunoglobulin free light chain testing, and serum and urine immunofixation in AL amyloidosis.

The endomyocardial biopsy can be valuable in doubtful cases, when non-invasive tests are inconclusive. Unfortunately, in idiopathic RCM it often demonstrates non-specific findings such as myocyte hypertrophy, interstitial and/or endocardial fibrosis. Furthermore, periprocedural risks in fragile affected children should be considered (50).

Restrictive cardiomyopathy should be distinguished from CP, since the two diseases have different treatments and outcomes. In some cases—namely, radiation induced cardiac disease—restriction and constriction may coexist in the same patient, making final diagnosis even more challenging. In Table 1 the main instrumental features of each condition are summarized.

**Table 1:** Differential diagnosis of constrictive pericarditis and restrictive cardiomyopathy.

	**Constrictive pericarditis**	**Restrictive cardiomyopathy**
**Clinical examination**	Kussmaul's sign, usually present	Kussmaul's sign, may be present
	Pulsus paradoxus (may be present)	Pulsus paradoxus (infrequent)
	Pericardial knock	S3; Systolic murmur due to mitral and tricuspidal regurgitaion
**Echocardiogram**		
Pericardial appearance	Thickened/bright	Normal
Atrial size	Minor enlargement	Major enlargement
Septal motion	Respiratory shift	Normal
Mitral inflow respiratory variation	Usually present (>25%)	Absent
TDI septal S' wave, cm/s	>5	<5
Speckle tracking	↓ Circumferential strain	↓ Radial and longitudinal strain
**Biomarkers**		
NT-proBNP	Normal or slightly abnormal	Abnormal
**Cardiac catheterization**		
Right and left ventricular end-diastolic pressures comparison	Equal or ≤ 5 mm Hg	Usually left > right
Dip-plateau waveform	Typically present	Can be present
**CT scan/MRI**		
Pericardial thickening	Present	Absent

## Management

Current medical therapy for RCM is primarily supportive and is in large part limited to diuretics in patients with signs and symptoms of systemic or pulmonary venous congestion. The International Society for Heart and Lung Transplantation (ISHLT) guidelines for the management of pediatric heart failure published in 2014 recommend in class I the diuretic therapy to establish a clinically euvolemic state, with a close monitoring of renal function and blood pressure during initiation and up-titration (67). Diuretic therapy reduces signs of systemic congestion, with beneficial effect on symptoms as dyspnea, fatigue, peripheral edema, and cough. However, excessive diuresis should be avoided because these patients are sensitive to alterations in preload. Furthermore, diuretics can mask an underlying PH or increased biventricular filling pressures, therefore this aspect must be considered before performing a hemodynamics invasive assessment (particularly prior to candidacy to heart transplantation). Restrictive cardiomyopathy has a unique pathophysiology: small ventricular cavity dimensions and rapid increase of filling pressures significantly compromise stroke volume (according to pressure-volume loop). Therefore, cardiac output is strongly influenced by heart rate, which must be kept at relatively high values to guarantee adequate systemic perfusion. For these reasons β-blockers and calcium channel blockers are not currently recommended in pediatric RCM, unless for a different indication (67). Angiotensin converting enzyme inhibitors and angiotensin receptor blockers may be considered if coexisting systemic arterial hypertension is present (class IIb recommendation). Similarly, digoxin (unless for rate control of atrial arrhythmias), intravenous inotropes (such as dopamine, dobutamine, and epinephrine), and pulmonary vasodilators (prostaglandins and endothelin receptor antagonists to treat secondary PH) are generally not recommended (Class III).

Although atrial thrombosis has been linked to atrial fibrillation, abnormal hemodynamics, and to a possible hypercoagulable state in adults with RCM, dedicated studies in pediatric populations are lacking (68). The incidence of intracardiac thrombus in the reviewed literature ranges from 0 to 42%, with rates of embolism between 12 and 33%. The risk of embolism appears to be much greater in children with RCM than to DCM; therefore, antithrombotic or anticoagulation therapy should be considered at the time of diagnosis (47, 69). However, there are no studies comparing the effectiveness of antiplatelet agents, vitamin K antagonists or enoxaparin in preventing embolism in children with RCM (70).

Conduction system disease recognize different etiologies, such as ischemic injury of the atrio-ventricular node and His-Purkinje system, mechanical stretching due to atrial and ventricular dilation or genetically determined mechanisms (71–73). In addition, prolonged PR interval and wide QRS complex at ECG were associated with acute cardiac event (49). The 2014 ISHLT guidelines recommend in Class I permanent PM for advanced second- or third-degree atrioventricular block associated with ventricular dysfunction. On the basis of available data, the presence of conduction system disease should trigger increased surveillance through baseline ECG and ECG Holter monitoring with ST-segment analysis together with a routine screening for research of clinical (chest pain or syncope) and instrumental ischemia (ST-segment variations). Some Authors suggest in the presence of PR prolongation, QRS widening, and left bundle-branch block particular attention and to consider prophylactic pacing, eventually as part of an implantable-cardioverter defibrillators (ICD) system (48). However, it should be noticed that there are no studies documenting the efficacy of defibrillator systems in large pediatric cohorts, and case to case evaluation, assessing the individual risk factors (including the specific etiology) should be carried out, in order to avoid inappropriate and detrimental therapies (49). At moment, without unanimous criteria, ICD should be considered in the subset of pediatric RCM patients with evidence of ischemia and ventricular arrhythmia, where also β-blockers may be beneficial (48). In a single center experience of pediatric patients with RCM, 40% of them with an ICD or PM, device therapies were relatively rare and inappropriate therapies were exceedingly rare (6).

These gaps in evidence are partly to ascribe to the low prevalence of RCM and its poor prognosis, with half patients dying or being referred to cardiac transplantation within 3 years from the diagnosis (74). Medical treatment has not shown any significant long term benefit and cardiac transplantation is the only effective therapy with survival rates between 70 and 60% at 5 and 10 year, respectively, in children listed for heart transplant (75). Orthotopic heart transplantation is preferred to heart-lung or heterotopic heart transplant and has a better survival rate than the other two options (76). In 2012 Singh et al. analyzed 1,436 children <18 years of age with a diagnosis of cardiomyopathy listed for heart transplant in the United States between 2004 and 2010, of which 167 with RCM. In adjusted analysis, children with non-DCM (83%) had a higher risk of wait-list mortality only if supported by a ventilator at listing. Post-transplant 30-days and at 1-year survival were similar between children with dilated and non-DCM (*p* = 0.17) (77). In another cohort of children with RCM from the American Pediatric Cardiomyopathy Registry database, about two thirds of children had a pure RCM phenotype, and the rest had a mixed RCM/HCM phenotype. Rate of survival at 5 year was 20 and 28%, respectively, but patients with pure RMC phenotype underwent heart transplantation more frequently (58 vs. 30%) (5).

The majority of deaths in children awaiting heart transplantation is due to progressive heart and multi-organ failure. Hence, in this context, mechanical circulatory support (MCS), as bridge to transplantation or candidacy, may be a precious weapon. At the moment the experience with MCS in children is quite limited, and this is particularly true for RCM. The most used pediatric long-term support device, is the pneumatically driven, pulsatile EXCOR^®^ Berlin Heart, with a variety of pump sizes, covering almost all pediatric patients, and the only long-term device for neonates and infants approved in Europe and USA. Few case series of successful bridging to cardiac transplantation with the Berlin EXCOR^®^ left ventricular assist device (LVAD) have been described (78, 79). Conventional LV apical cannulation of a non-compliant left ventricle often results in insufficient drainage and poor pump performance, with residual high left atrial pressures and consequent pulmonary congestion. Left atrial cannulation (such as in EXCOR^®^ Berlin Heart) is therefore an interesting option in patient with small ventricular cavity, preserved systolic function, and enlarged atria (80). A recent review of the American registry of EXCOR^®^ Berlin Heart implantation in pediatric patients affected by RCM showed a survival rate of 50%, which is significantly less than that of the overall EXCOR ^®^ pediatric population (75%) (81). Primary causes of death included stroke, infection, acidosis, multisystem organ failure, and bleeding. It is of note that these patients tended to be sicker than the whole population (INTERMACS class 1 and with ECMO support) and this can explain at least partly, the worst outcome. In summary, long-term MCS implantation is a high-risk procedure that can be considered in advanced stages of the disease as bridge to transplantation or to candidacy by improving hemodynamics, with the reduction of post-capillary PH. Further studies are needed to determine the best timing for the procedure and the best anticoagulant strategy to reduce the risk of thromboembolic events that are the main cause of adverse outcome in these patients.

**Figure 7:**
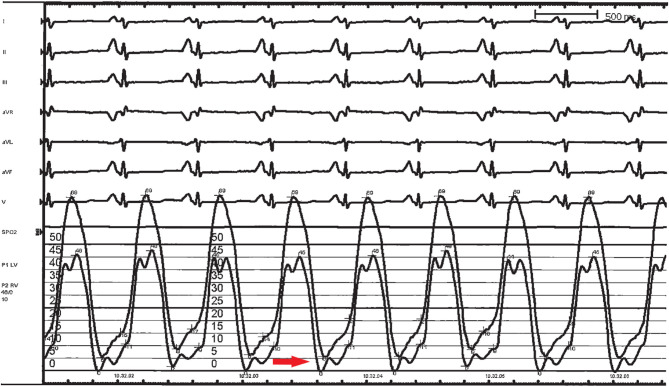
Left ventricular (LV) and right ventricular (RV) hemodynamic pressure tracings in a 3-year-old girl affected by restrictive cardiomyopathy. End-diastolic filling pressures are elevated, with LV values higher than those in RV, and a “square root” sign is present on both tracings (red arrow).

Considering all these major concerns about the poor efficacy of medical treatment, the identification of the right time to list for heart transplantation a patient affected by RCM during the clinical follow-up becomes both challenging and crucial. Although some clinical and instrumental factors such as pulmonary congestion at diagnosis, severe left atrial dilation, or increased ventricular wall thickness have been identified as potential predictors of poor prognosis, there are no established criteria for listing and the decision is often dependent on individual experienced centers (82). A multi-modal instrumental approach is essential, particularly based on a regular assessment of right heart hemodynamics with or without use of inotropic agents and vasodilators, to detect at the proper time the development of irreversible PH. Some pediatric institutions consider the development of PH an indication for listing, regardless of heart failure symptoms. Accordingly, right heart catheterization is mandatory at first evaluation, since up to 50% of patients have PH at diagnosis (47, 83). The flow-chart illustrated in Figure 7 summarizes the experienced approach for pediatric RCM, developed in our tertiary center, with a dedicated program for pediatric cardiomyopathies and pediatric heart transplantation.

## Conclusion

Pediatric RCM is a rare disorder, due to a large heterogeneous group of causes. As a result of its poor prognosis, RCM contributes disproportionately to mortality in children with cardiomyopathy, being heart transplantation the only effective treatment. Therefore, early referral to a third-level cardiomyopathy center is warranted for careful observation, to avoid the development of irreversible PH and to avoid a delay in listing for heart transplantation when indicated.

## Author Contributions

All authors listed have made a substantial, direct, and intellectual contribution to the work and approved it for publication.

## Conflict of Interest

The authors declare that the research was conducted in the absence of any commercial or financial relationships that could be construed as a potential conflict of interest.

## Publisher's Note

All claims expressed in this article are solely those of the authors and do not necessarily represent those of their affiliated organizations, or those of the publisher, the editors and the reviewers. Any product that may be evaluated in this article, or claim that may be made by its manufacturer, is not guaranteed or endorsed by the publisher.
